# Phototherapy modifies hematologic markers without inducing inflammation in neonates: a retrospective observational study

**DOI:** 10.1007/s00431-025-06240-3

**Published:** 2025-06-06

**Authors:** Ceren Can, Şahin Hamilçıkan, Yakup Karakurt, Emrah Can

**Affiliations:** 1 Department of Pediatric Immunology and Allergy, University of Health Sciences, Istanbul, Turkey; 2 Department of Pediatrics, University of Health Sciences, Istanbul, Turkey; 3 Department of Pediatrics, University of Health Sciences, Bağcılar Training and Research Hospital, Istanbul, Turkey

**Keywords:** Phototherapy, Oxidative stress, Neonatal hyperbilirubinemia, Hematologic markers, Preterm infants

## Abstract

Phototherapy is the standard treatment for neonatal hyperbilirubinemia. However, its impact on hematologic indices related to oxidative stress and systemic inflammation, particularly in preterm infants, remains insufficiently understood. In this retrospective observational study, 96 neonates who received phototherapy were evaluated. Hematologic ratios including red cell distribution width to platelet count (RDW/PLT), RDW to lymphocyte count (RDW/LF), and platelet distribution width to plateletcrit (PDW/PCT) were analyzed. Systemic inflammatory markers such as neutrophil-to-lymphocyte ratio (NLR), platelet-to-lymphocyte ratio (PLR), systemic inflammatory index (SI), systemic immune inflammation index (SIRI), and pan-inflammatory value (PIV) were also compared before and after treatment using Wilcoxon signed-rank tests. Phototherapy was associated with an increase in RDW/PLT from a median of 0.11 to 0.14 (*p* = 0.0007, 95% CI 0.02 to 0.05) and in RDW/LF from 2.98 to 3.62 (*p* = 0.0005, 95% CI 0.50 to 0.80), as well as a decrease in PDW/PCT from 42.1 to 36.4 (*p* = 0.0001, 95% CI − 8.0 to − 4.5). No significant changes were observed in systemic inflammatory markers (*p* greater than 0.05 for all). *Conclusion*: Phototherapy induces localized hematologic changes that may reflect oxidative activity, without provoking systemic inflammation. RDW/PLT and RDW/LF may serve as accessible indicators of oxidative response. These findings support the hematologic safety of phototherapy in term and near-term neonates, while further research is needed in extremely preterm infants due to their increased susceptibility.

**Table:** 

**What is Known:** • *Phototherapy is an effective and widely used treatment for neonatal jaundice* • *Experimental studies suggest that it may influence oxidative stress pathways* **What is New:** • *This study demonstrates that phototherapy alters RDW-based hematologic ratios without activating systemic inflammation* • *RDW/PLT may serve as a practical early marker of oxidative stress response in neonates, reinforcing the safety of phototherapy while raising questions for preterm care*

**What is Known:**

• *Phototherapy is an effective and widely used treatment for neonatal jaundice*

• *Experimental studies suggest that it may influence oxidative stress pathways*

**What is New:**

• *This study demonstrates that phototherapy alters RDW-based hematologic ratios without activating systemic inflammation*

• *RDW/PLT may serve as a practical early marker of oxidative stress response in neonates, reinforcing the safety of phototherapy while raising questions for preterm care*

## Introduction

Neonatal hyperbilirubinemia affects about 60% of term and up to 80% of preterm infants in their first week of life [1]. While typically benign, severe cases can lead to serious conditions like acute bilirubin encephalopathy or kernicterus, risking permanent neurological damage [2]. Phototherapy, which breaks down bilirubin into excretable isomers, is the standard treatment for managing this condition [3]. However, growing evidence suggests it may also induce oxidative stress via light-mediated production of reactive oxygen species, potentially influencing systemic physiology beyond bilirubin metabolism [3–5].

Experimental research has shown that phototherapy’s light exposure can generate reactive oxygen species (ROS), possibly affecting red blood cell membranes, platelet function, and inflammatory pathways [5, 6]. This is especially relevant for preterm infants, whose immature skin and antioxidant systems may heighten their susceptibility. Notably, extremely preterm neonates have significantly thinner skin, which may allow deeper light penetration during phototherapy [7]. A recent study emphasized that light penetration might extend deeper in extremely preterm babies, increasing their risk of oxidative and inflammatory damage [7]. Interestingly, bilirubin can act as an antioxidant at low levels [8], though excess amounts may promote oxidative stress.

Hematologic markers such as red cell distribution width (RDW), plateletcrit (PCT), mean platelet volume (MPV), and derived ratios like RDW to platelet count (RDW/PLT), RDW to lymphocyte count (RDW/LF), and platelet distribution width to plateletcrit (PDW/PCT) are gaining recognition as accessible indicators of inflammation and oxidative stress in neonates [9–11]. These are increasingly applied in neonatology, particularly for diagnosing sepsis, though their role in conditions like intraventricular hemorrhage (IVH) is less established [10, 12].

Systemic inflammatory markers, including the neutrophil-to-lymphocyte ratio (NLR), platelet-to-lymphocyte ratio (PLR), systemic inflammatory index (SI), systemic immune-inflammatory response index (SIRI), and pan-inflammatory value (PIV), help assess immune status in newborns, particularly in early-onset sepsis and intraventricular hemorrhage [10, 12, 13]. Whether phototherapy affects these markers remains an open question.

This study aims to explore how phototherapy influences hematologic and inflammatory indices in neonates, focusing on RDW/PLT, RDW/LF, and PDW/PCT ratios, as well as systemic markers, in term and near-term infants. We also consider the clinical implications, particularly for preterm infants who may be more at risk.

## Methods

This retrospective observational study was carried out at the Neonatology Department of the University of Health Sciences, Istanbul, from January 2023 to December 2024. We included neonates treated with phototherapy for unconjugated hyperbilirubinemia who had complete blood count data before and after treatment. Eligible participants were at least 34 weeks gestation, met Bhutani nomogram thresholds for treatment, as recommended by the American Academy of Pediatrics guidelines [1, 14], and had no congenital or hemolytic disorders.

We excluded infants with confirmed or suspected infections, hemolytic conditions (e.g., ABO or Rh incompatibility), glucose-6-phosphate dehydrogenase (G6PD) deficiency, congenital anomalies, or blood samples taken before 72 h of life. Extremely preterm infants were not included. To reduce bias, we only analyzed cases with consistent clinical and laboratory records.

The study was approved by the Institutional Review Board (2024/12/12/098) and followed the Declaration of Helsinki guidelines.

All infants received phototherapy via LED devices (Novos Blisphere 360, Neloled Maxi) emitting light at 450–470 nm with an irradiance of 20–25 µW/cm^2^/nm. Treatment ended when bilirubin levels dropped below the threshold for the infant’s postnatal age.

Venous blood samples were collected within 12 h before and after phototherapy. Standard hematologic parameters (RDW, PLT, LF, PDW, PCT, NEU, and MON) were retrieved from electronic medical records. Derived indices were calculated as follows:

- RDW/PLT = RDW/platelet count
- RDW/LF = RDW/lymphocyte count
- PDW/PCT = PDW/plateletcrit
- NLR = NEU/LF
- PLR = PLT/LF
- SI = (PLT × NEU)/LF
- SIRI = (NEU × MON)/LF
- PIV = (PLT × NEU × MON)/LF [18–20]

### Statistical analysis

We used SPSS version 25.0 (IBM Corp., Armonk, NY) for all statistical analyses. The Shapiro–Wilk test was used to assess the normality of distributions. Due to non-normal distribution of most variables, pre- and post-phototherapy comparisons were conducted using the Wilcoxon signed-rank test. A *p*-value < 0.05 was considered statistically significant. Sample size was calculated using G*Power (v3.1), assuming an effect size of 0.3, *α* = 0.05, and 80% power, requiring a minimum of 89 participants.

## Results

**Fig. 1:**
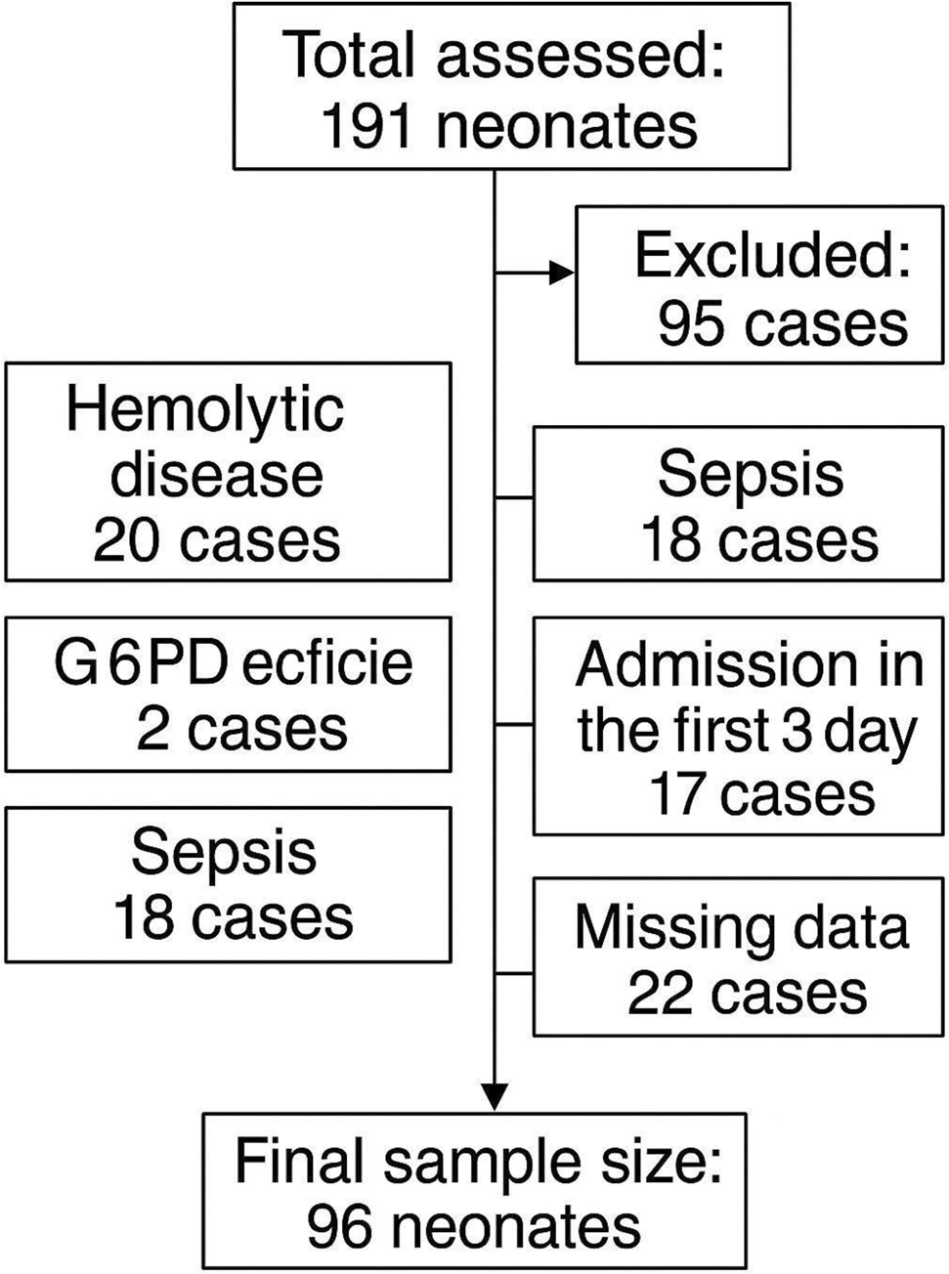
Flowchart of screening (*n* = 191), exclusions (*n* = 95), and final cohort (*n* = 96)

We analyzed data from 96 neonates who met the inclusion criteria, as summarized in the flowchart (Fig. 1). Among the 191 assessed neonates, 95 were excluded due to hemolytic disease (*n* = 20), systemic illness (*n* = 16), G6PD deficiency (*n* = 2), sepsis (*n* = 18), admission within the first 3 days (*n* = 17), or missing data (*n* = 22).

The baseline demographic and clinical characteristics of the final cohort are detailed in Table 1. The mean gestational age was 37.60 ± 1.39 weeks, and the mean birth weight was 3070.87 ± 537.87 g. The cohort included 54% male infants, and 60% were delivered via cesarean section. The mean length of hospital stay was 3.91 ± 1.44 days, and the average bilirubin level at the onset of phototherapy was 18.28 ± 3.92 mg/dL.

**Table 1:** Baseline demographic and clinical characteristics of the study cohort (*n* = 96)

Variable	Mean ± SD	Range
Gestational age (weeks)	37.60 ± 1.39	34–42
Birth weight (g)	3070.87 ± 537.87	1980–4840
Gender (% male)	54%	N/A
Delivery method (NSD/C-section)	40%/60%	N/A
Length of stay (days)	3.91 ± 1.44	2–7
Weight loss (%)	5.78 ± 4.66	15–19
Bilirubin level (mg/dL)	18.28 ± 3.92	11–21
Albumin level (g/dL)	4.13 ± 0.21	2.14–4.32

Table 2 summarizes the changes in hematologic indices before and after phototherapy. Significant increases were observed in RDW/PLT (0.11 → 0.14; *p* = 0.0007) and RDW/LF (2.98 → 3.62; *p* = 0.0005) ratios. In contrast, the PDW/PCT ratio significantly decreased from 42.1 to 36.4 (*p* = 0.0001). Other parameters, including RDW, MPV, PDW, PCT, NLR, PLR, SI, SIRI, and PIV, showed no statistically significant change (*p* > 0.05).

**Table 2:** Pre- and post-phototherapy hematologic indices. Data are shown as median (IQR); comparisons made using Wilcoxon signed-rank test

Variable	Pre (median ± IQR)	Post (median ± IQR)	*p*-value	Wilcoxon statistic
RDW	15.77 ± 0.89	18.61 ± 1.51	NS	304.5
RDW/PLT	0.11 ± 0.03	0.14 ± 0.04	0.0007	1089.0
RDW/LF	2.98 ± 0.50	3.62 ± 0.60	0.0005	1071.0
MPV	11.22 ± 9.30	10.42 ± 1.49	NS	751.5
PCT	0.34 ± 0.12	0.61 ± 1.80	NS	705.5
PDW	16.49 ± 1.78	16.65 ± 0.28	NS	1147.5
NLR	0.90 ± 0.61	0.90 ± 0.74	NS	1672.0
PLR	70.22 ± 28.84	72.90 ± 33.76	NS	1818.0
PDW/PCT	42.1 ± 8.0	36.4 ± 6.0	0.0001	982.0
SI	291.00 ± 241.46	366.31 ± 421.39	NS	1761.0
SIRI	1.17 ± 0.98	1.24 ± 1.56	NS	1630.0
PIV	393.06 ± 381.59	463.52 ± 507.81	NS	1800.0

**Fig. 2:**
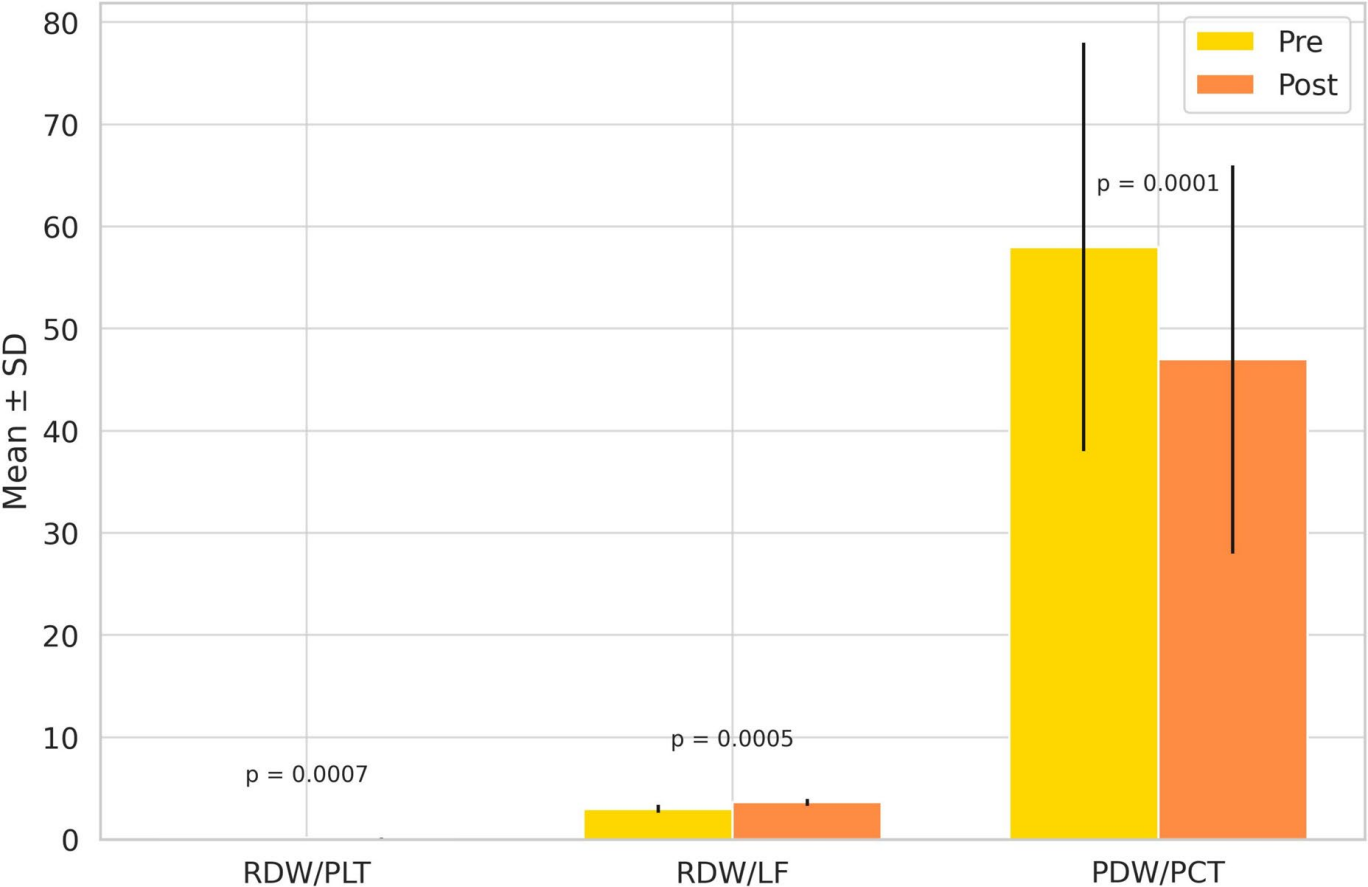
RDW/PLT, RDW/LF, and PDW/PCT before (yellow) and after (orange) phototherapy. Bars show mean ± SD. Paired *t*-test;* p* < 0.01

These findings are visualized in Fig. 2, which shows pre- and post-treatment values as bar plots with mean ± SD. Although Wilcoxon tests were used, data are presented as mean ± SD for visual clarity.

**Fig. 3:**
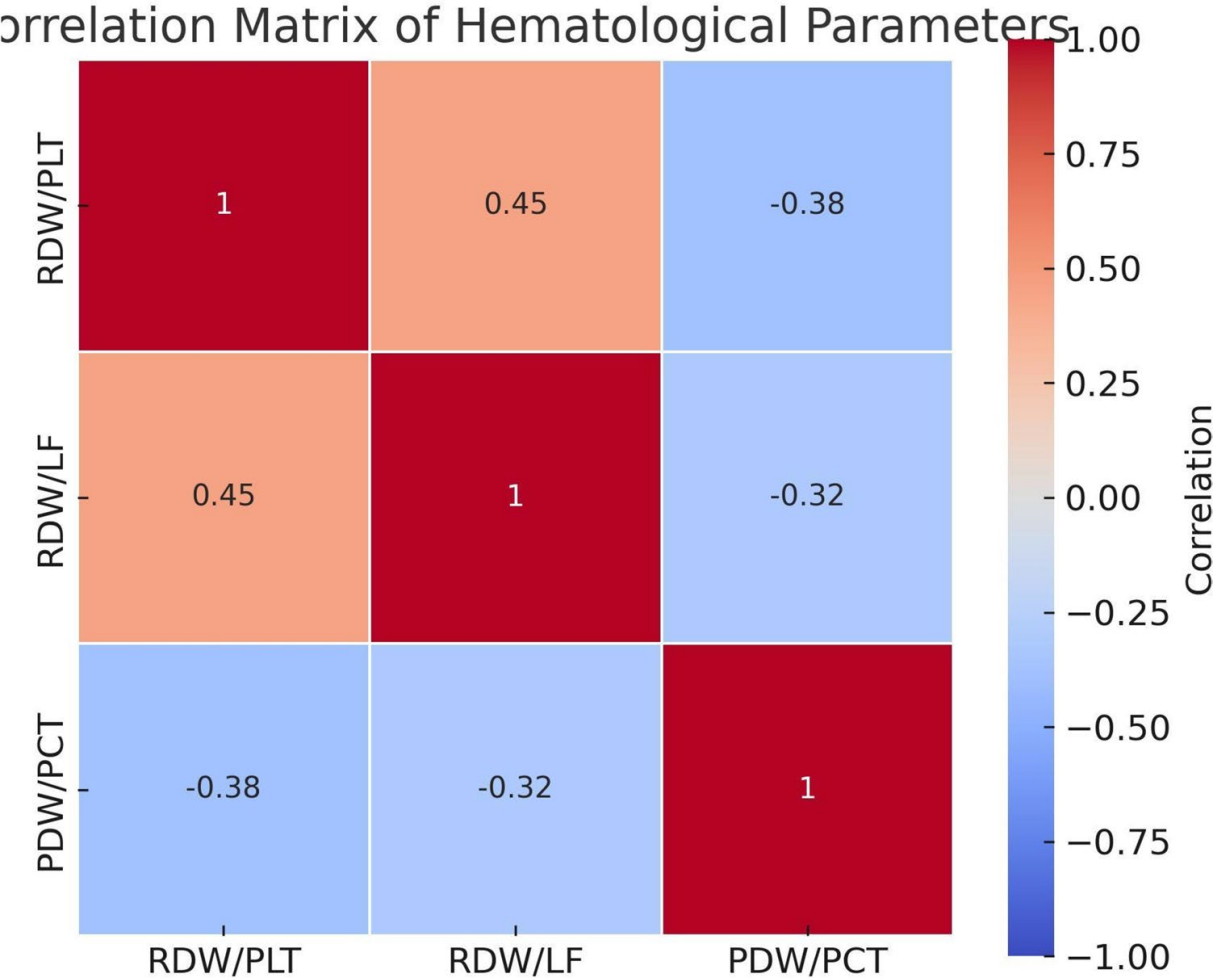
Correlation matrix of hematologic ratios post-phototherapy. Pearson correlation coefficients (*r*) are shown in each cell. Positive correlations are shaded in red and negative in blue*.* All shown correlations were statistically significant at *p* < 0.01

Additionally, Fig. 3 presents a correlation matrix of hematologic ratios post-phototherapy. RDW/PLT and RDW/LF were moderately correlated (*r* = 0.45), while PDW/PCT was negatively correlated with both RDW/PLT (*r* = − 0.38) and RDW/LF (*r* = − 0.32), suggesting distinct response dynamics.

## Discussion

Our findings indicate that phototherapy alters certain hematologic ratios linked to oxidative stress, such as RDW/PLT and RDW/LF, without triggering systemic inflammation in neonates. This aligns with prior experimental evidence suggesting that phototherapy, while effective for bilirubin reduction, may cause localized oxidative stress through photochemical reactions [8, 15].

The rise in RDW-based ratios could point to temporary red blood cell membrane instability or mild hemolysis, as ROS can damage erythrocytes and increase anisocytosis [4, 5]. The drop in PDW/PCT further suggests platelet involvement in this oxidative response, consistent with earlier findings that platelet indices may respond to subclinical stress and inflammation [9]. Yet, the stability of systemic markers like NLR, PLR, SIRI, SI, and PIV indicates that phototherapy does not broadly activate inflammation in term and near-term infants, supporting previous findings that cytokine levels and lymphocyte subsets remain unaffected by phototherapy [16, 17]. The absence of clinical issues like sepsis in our cohort supports the idea that these changes are likely a normal response rather than a pathological one, offering reassurance about phototherapy’s safety.

This study adds to the growing body of evidence on phototherapy-related oxidative stress. While previous research has noted changes in antioxidant capacity and lipid peroxidation [18], few have examined hematologic markers as early signals. The lack of inflammatory marker shifts is particularly encouraging, given phototherapy’s widespread use in preterm infants, where about 80% may require it.

However, extremely preterm infants might respond differently. A recent study by Foligno et al. [7] highlighted their thinner skin, which may allow deeper light penetration and increase oxidative burden. Preterm neonates with thinner skin show lower transcutaneous bilirubin and higher predicted mortality, suggesting enhanced light penetration and possibly greater systemic oxidative impact [7, 19, 20]. With immature antioxidant systems and lower glutathione levels, these infants could be more prone to complications like bronchopulmonary dysplasia or retinopathy of prematurity. Since our study excluded those under 34 weeks, these findings may not apply to this group. Importantly, emerging evidence suggests that the skin immaturity of extremely preterm infants may play a pivotal role in their vulnerability to oxidative stress. Foligno et al. [7] recently demonstrated that thinner skin in NICU-admitted preterm neonates was associated with both lower transcutaneous bilirubin levels and higher predicted mortality, independent of gestational age or serum bilirubin. These findings imply that reduced dermal thickness may permit deeper light penetration during phototherapy, potentially exposing internal tissues to photochemical injury. While our study did not include infants below 34 weeks of gestation, this mechanism may partially explain the paradoxical outcomes observed in more immature populations. Further research targeting this group is warranted to elucidate whether tailored phototherapy strategies based on skin characteristics might improve safety.

Key limitations include the absence of a control group, which limits causal inference and leaves room for natural variation to explain some of the observed changes. Additionally, we lack longitudinal data to determine whether these hematologic shifts persist or are linked to clinical outcomes such as anemia or thrombocytopenia. To address these issues, future prospective studies should incorporate matched control groups and extended follow-up to clarify the temporal dynamics and clinical relevance of these markers.

Nevertheless, the practical utility of ratios like RDW/PLT lies in their simplicity and bedside accessibility. These indices could serve as surrogate markers for oxidative changes during neonatal care, particularly in low-resource settings where advanced oxidative stress assays are not available. The association between increased RDW and risks such as anemia further suggests that routine monitoring might enable earlier detection and intervention in at-risk neonates.

## Conclusion

In summary, phototherapy in late preterm and term neonates was associated with rises in RDW-based oxidative stress ratios without a corresponding increase in systemic inflammation. The absence of clinical deterioration suggests these shifts reflect a temporary response to treatment rather than an underlying disease process. Since RDW to platelet and RDW to lymphocyte ratios are cost-effective and routinely available, they may serve as practical indicators of subtle oxidative changes in low-resource settings. Future randomized studies including an appropriate control group may help determine whether these alterations are truly specific to phototherapy. Additionally, studies in extremely preterm infants could clarify how long these changes persist and whether closer monitoring can improve neonatal outcomes.

## Data Availability

No datasets were generated or analysed during the current study.

## Abbreviations

MPV: Mean platelet volume
NLR: Neutrophil-to-lymphocyte ratio
PCT: Plateletcrit
PDW: Platelet distribution width
PLR: Platelet-to-lymphocyte ratio
RDW: Red cell distribution width
RDW/LF: RDW to lymphocyte
RDW/PLT: RDW to platelet
SI: Systemic ınflammatory ındex
SIRI: Systemic ımmune-ınflammatory response ındex
PIV: Pan ınflammatory ındex
